# Round robin study of formalin-fixed paraffin-embedded tissues in mass spectrometry imaging

**DOI:** 10.1007/s00216-018-1216-2

**Published:** 2018-07-03

**Authors:** Achim Buck, Bram Heijs, Birte Beine, Jan Schepers, Alberto Cassese, Ron M. A. Heeren, Liam A. McDonnell, Corinna Henkel, Axel Walch, Benjamin Balluff

**Affiliations:** 1 Research Unit Analytical Pathology, Helmholtz Zentrum München, 85764 Oberschleißheim, Germany; 2 Center for Proteomics and Metabolomics, Leiden University Medical Center, 2333 ZA Leiden, The Netherlands; 3 Medizinisches Proteom-Center, Ruhr-Universität Bochum, 44801 Bochum, Germany; 4 Leibniz-Institut für Analytische Wissenschaften – ISAS-e.V, 44139 Dortmund, Germany; 5 Department of Methodology and Statistics, Faculty of Psychology and Neuroscience, Maastricht University, 6200 MD Maastricht, The Netherlands; 6 The Maastricht MultiModal Molecular Imaging Institute (M4I), Maastricht University, Universiteitssingel 50, Pigeon Hole 57, P.O. Box 616, 6200 MD Maastricht, The Netherlands; 7 Fondazione Pisana per la Scienza ONLUS, 56017 Pisa, Italy; 8 Bruker Daltonik, Bremen, Germany

**Keywords:** Mass spectrometry imaging, Multicenter study, Formalin-fixed paraffin-embedded tissue, Peptides, Metabolites, Ring trial

## Abstract

Mass spectrometry imaging (MSI) has provided many results with translational character, which still have to be proven robust in large patient cohorts and across different centers. Although formalin-fixed paraffin-embedded (FFPE) specimens are most common in clinical practice, no MSI multicenter study has been reported for FFPE samples. Here, we report the results of the first round robin MSI study on FFPE tissues with the goal to investigate the consequences of inter- and intracenter technical variation on masking biological effects. A total of four centers were involved with similar MSI instrumentation and sample preparation equipment. A FFPE multi-organ tissue microarray containing eight different types of tissue was analyzed on a peptide and metabolite level, which enabled investigating different molecular and biological differences. Statistical analyses revealed that peptide intercenter variation was significantly lower and metabolite intercenter variation was significantly higher than the respective intracenter variations. When looking at relative univariate effects of mass signals with statistical discriminatory power, the metabolite data was more reproducible across centers compared to the peptide data. With respect to absolute effects (cross-center common intensity scale), multivariate classifiers were able to reach on average > 90% accuracy for peptides and > 80% for metabolites if trained with sufficient amount of cross-center data. Overall, our study showed that MSI data from FFPE samples could be reproduced to a high degree across centers. While metabolite data exhibited more reproducibility with respect to relative effects, peptide data-based classifiers were more directly transferable between centers and therefore more robust than expected.

**Electronic supplementary material:**

The online version of this article (10.1007/s00216-018-1216-2) contains supplementary material, which is available to authorized users.

**Graphical abstract:**
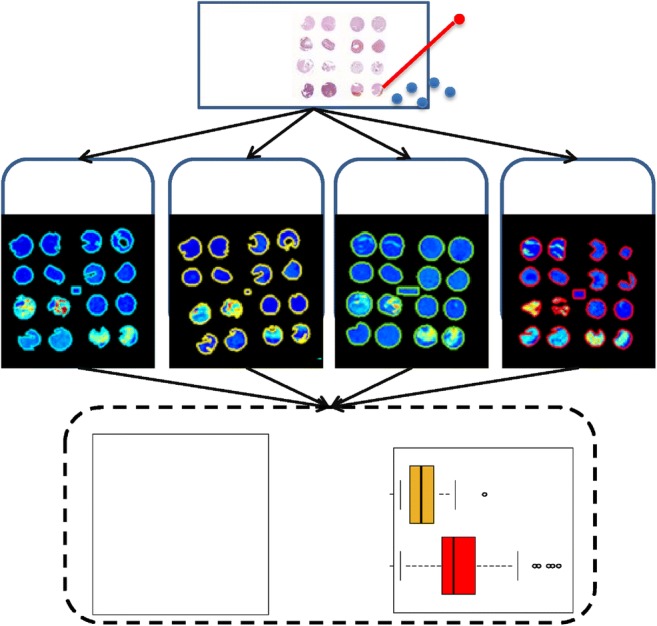
ᅟ

## Introduction

Mass spectrometry imaging (MSI) is a technology, which allows the investigation of spatial distributions of ionized molecules from surfaces [1]. The spatial character of MSI has especially proven useful in biomedical research to unscramble the cellular and morphological complexity of biological tissue specimens [2]. This has led in many studies to the finding of disease- and cell-type-specific molecular profiles in tissue-related pathologies [3]. Frequently, these profiles are ascribed diagnostic or prognostic potential in a prospective clinical setting [4]. But results with translational ambition have to be examined sufficiently to prove a robust and reproducible application in large patient cohorts and across different centers before they can become “bedside” [5].

Few biomedically oriented multicenter MSI studies have already been conducted on fresh-frozen tissues [6, 7]. Dekker *et al*., for instance, reported the reproducibility of three out of four protein markers for stromal activation in breast cancer between two centers [7]. With respect to the clinically more common formalin-fixed paraffin-embedded (FFPE) tissues, only one study has analyzed samples from various centers albeit in a centralized way [8]. While the analysis of 102 tissues from 11 countries found MSI to provide a better prediction for clinical outcome than histopathology [8], the centralized design of the study overlooked the potential interlaboratory technical variation for future on-site implementations. It is therefore important to get an understanding of the degree of intercenter technical variation and its effect on masking biological effects.

This is addressed by a round robin design, which is usually the first step toward clinical multicenter studies [9]. A round robin aims for standardization and quantification of interlaboratory variation given similar or identical samples, experimental protocols, and instrumentation [10, 11]. A bicenter round robin study on frozen tissue has already proven the reproducibility (intercenter) and repeatability (intracenter) of desorption electrospray ionization MSI [12].

In the presented study, the first round robin MSI study on FFPE tissues with the goal to investigate the consequences of inter- and intracenter technical variation on masking biological effects was performed. A total of four centers with similar or equal MS instrumentation (Bruker Ultraflex II, III, or UltrafleXtreme) and sample preparation equipment (SunChrom SunCollect sprayer for matrix and trypsin application) were involved in this study. FFPE tissue has been chosen to match clinical practice and the ease of sample distribution for future multicenter studies. For the purpose of the study, a multi-organ tissue microarray (TMA) was constructed containing samples from eight different mouse organs, which enabled investigating various biological differences. Given the possibility to extract peptide and metabolite information from FFPE tissues, the study was performed for both molecular classes using slightly adapted versions of recently published protocols by two of the participating centers [13, 14].

Given this scenario, this study will investigate for each of the two molecular classes the degree of reproducibility for univariate statistical testing and the applicability of univariate or multivariate classifiers across different centers.

## Material and methods

### Material and logistics

**Fig. 1:**
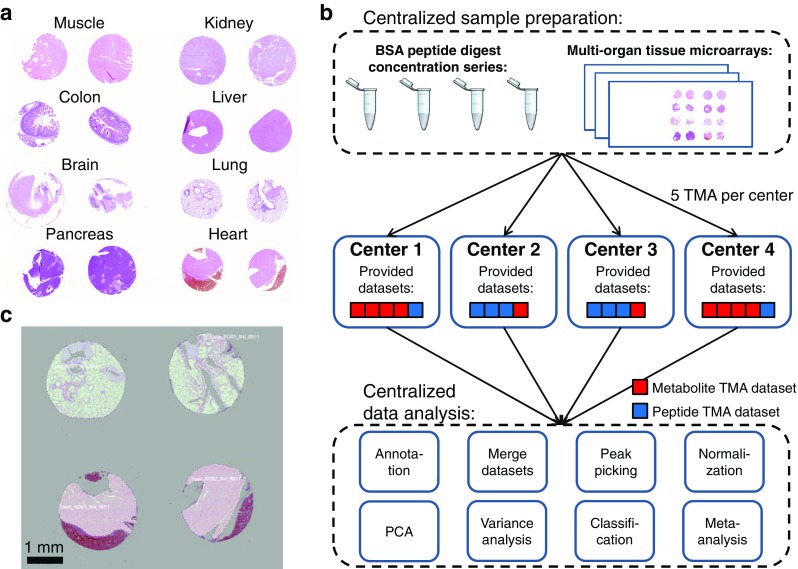
This round robin study made use of a tissue microarray (TMA), which contained 16 needle core biopsies from eight different organs and two different wild-type mice (**a**). Twenty consecutive sections of this TMA were distributed in a randomized order to each of the four participating centers (each center receives 5 sections), together with a concentration series of a bovine serum albumin (BSA) digest (**b**, top). Centers 2 and 3 were required to measure at least one of the samples on a metabolite level and three on a peptide level, and centers 1 and 4 vice versa (**b**, middle). The data was then collected from all centers and analyzed centrally (**b**, bottom). The preprocessing of the data also included a centralized manual annotation of the tissue (**c**)

A multi-organ tissue microarray was constructed by assembling 16 two-millimeter-sized tissue punches from formalin-fixed paraffin-embedded tissues of eight organs (brain, colon, heart, kidney, liver, lung, pancreas, and skeletal muscle) from two wild-type mice (Fig. 1a). After sacrifice, the rodent tissue samples (4 mm thick) were fixed in 4% (vol/vol) neutral-buffered formalin (Sigma-Aldrich, Germany) at room temperature, routinely prepared for paraffin embedding with an automatic processor (Tissue-Tek® VIPTM, Sakura, Europe), and finally embedded in paraffin wax. Consecutive 6-μm sections were made on a paraffin microtome (HM325, Microm, Germany) and placed separately on previously poly-L-lysine-coated indium-tin-oxide glass slides (Bruker Daltonik, Bremen, Germany) as described before [14]. Each of the four participating centers (affiliations 1, 2, 4, and 6, and further anonymized to centers 1, 2, 3, and 4) received randomized five virtually consecutive sections with the task to perform the experiments within 3 weeks after reception. Keeping one slide as backup, centers 2 and 3 had to perform at least one metabolite and three peptide experiments and centers 1 and 4, at least one peptide and three metabolite experiments (Fig. 1b and Table 1).

**Table 1:** Technical equipment and provided datasets of consortium members

Center	Delivered datasets		Instrumentation			
	Metabolites	Peptides	Mass spectrometer	Spray robot	Antigen retrieval system	Optical slide scanning system
1	4	1	Ultraflex III, Bruker Daltonics	SunCollect, SunChrom	Antigen Retrieval in 97 °C water bath	Mirax Desk, Zeiss
2	1	3	UltrafleXtreme, Bruker Daltonik	SunCollect, SunChrom	Antigen Retriever 2100, Aptum Biologics	Mirax Desk, Zeiss
3	1	3	UltrafleXtreme, Bruker Daltonik	SunCollect, SunChrom	Antigen Retriever 2100, Aptum Biologics	IntelliSite Ultra-Fast Scanner, Philips
4	4	1	Ultraflex II, Bruker Daltonik	SunCollect, SunChrom	Antigen Retriever 2100, Aptum Biologics	Mirax Desk, Zeiss

### Sample preparation

The protocols for metabolite and tryptic peptide experiments were based on recently published protocols [13, 14], and the chemicals used are listed per center in Electronic supplementary material (ESM), Table S1. In both protocols, the tissue section was at first adhered to the slide by warming on a heating block at 60 °C for 1 h.

For metabolite experiments, paraffin was removed by two subsequent 8-min xylene washes followed by drying at room temperature and the application of fiducial markers. The matrix (10 mg/mL 9-aminoacridine hydrochloride monohydrate in 70% methanol) was prepared as described previously [14] and applied onto the sample with the SunCollect spraying system (SunChrom, Friedrichsdorf, Germany) using the following parameters: *x* = 0.5 mm; *y* = 2.0 mm; *z* = 20 mm; speed(*x*,*y*) = med(1) or 900 mm/min; flow rates: layers 1 to 3 at 10, 20, and 30 μL/min, respectively, and layers 4 to 8 at 40 μL/min.

For tryptic peptide experiments, paraffin was removed by two xylene washes for 5 and 10 min. Then the slides were washed twice for 2 min in 100% ethanol and twice for 5 min in ultrapure Milli-Q water. In centers 2, 3, and 4, the antigen retrieval was performed with 10 mM citric acid monohydrate at pH 6 as buffer in the Antigen Retriever 2100 (Aptum Biologics, Southampton, UK) according to the manufacturer’s instructions. Center 1 performed the antigen retrieval in a water bath at 97 °C in 10 mM citric acid buffer (pH 6) for 30 min. After the antigen retrieval, slides were allowed to cool to room temperature, followed by washing them twice for 1 min in ultrapure water and drying them for 15 min in a desiccator. The 0.02-μg/μL trypsin solution was prepared just before its application and sprayed with the SunCollect spraying system (SunChrom) with the following parameters: *x* = 0.5 mm; *y* = 1.0 mm; *z* = 25 mm; speed(*x*,*y*) = med(1) or 900 mm/min; flow rates: layers 1 to 15 at 10 μL/min. Incubation of the slide was done for 18 h at 37 °C in a saturated environment using an airtight box filled with 100 mL of 50% MeOH and 50% Milli-Q water. The next day, fiducial markers were placed on the slide before the matrix (7 mg/mL alpha-cyano-4-hydroxycinnamic acid in 50% acetonitrile/0.2% trifluoroacetic acid) was applied with the SunCollect sprayer (SunChrom) using the following parameters: *x* = 0.5 mm; *y* = 2.0 mm; *z* = 26 mm; speed(*x*) = low(7) or 490 mm/min; speed(*y*) = med(3) or 1055 mm/min; flow rates: layers 1 to 3 at 10, 20, and 30 μL/min, respectively, and layers 4 to 7 at 40 μL/min.

### Quality controls

Although all centers shared very similar instrumentation (Table 1), each MSI experiment was preceded by the measurement of a centrally distributed dilution series of a bovine serum albumin digest (Pierce™ BSA Protein Digest, # 88341, Thermo Fisher) in order to monitor potential intra- and intercenter differences in instrument performance. This concentration series was prepared centrally (ESM, Protocol S1) and shipped to all remaining partners on dry ice. Finally, each local laboratory mixed each dilution again 1:1 with their locally prepared matrix (7 mg/mL alpha-cyano-4-hydroxycinnamic acid in 50% acetonitrile/0.2% trifluoroacetic acid); 2 μL of each dilution was then pipetted onto an AnchorChip target plate (Bruker Daltonik) leading to absolute amounts in the spotted volume in the pico- to femtomole range.

For each droplet, 2500 spectra were acquired in random walk mode (50 spectra per step) over an area with a 500-μm diameter with the same settings as for the tryptic peptide MSI experiments (see below).

### Mass spectrometry imaging measurements

Before every measurement, the ion source was cleaned with isopropanol or ethanol. Metabolite measurements were performed in reflector mode with negative polarity, in the *m*/*z* range 200–1000 with suppression up to *m*/*z* 200, and a minimum sampling rate of 2 GS/s. As the spatial resolution was chosen to be 70 μm, the laser focus was set to medium. At each spot, 200 spectra were accumulated in random walk movement with 25 spectra per step. Spectra were smoothed (Gaussian filter, 2 cycles with a width of *m*/*z* 0.005) and baseline subtracted (tophat filter) on-the-fly via FlexAnalysis (Bruker Daltonik).

Peptide measurements were performed in positive mode, in the *m*/*z* range 800–4000 with suppression up to *m*/*z* 700, a minimum sampling rate of 2 GS/s, and a spatial resolution of 70 μm. At each spot, 500 spectra were accumulated in random walk movement with 50 spectra per step. Spectra were smoothed (Gaussian filter, 2 cycles with a width of *m*/*z* 0.02) and baseline subtracted (tophat filter) on-the-fly via FlexAnalysis (Bruker Daltonik).

Before the start of any measurement, the mass spectrometer was calibrated using phosphorus red, which was dissolved in acetone and spotted (1 μL) on the same glass slide into an area with matrix. Each center optimized the laser intensity in the very first experiment according to the subjective opinion of the local experimenter and left it constant for the rest of the project.

After measurements, the matrix was removed by a wash in 70% EtOH and stained for hematoxylin and eosin using local protocols. Optical images from the slides were obtained by local high-resolution slide scanners (Table 1) and coregistered to the MSI data in the FlexImaging software (Bruker Daltonik).

### Data management and preprocessing

Each participant uploaded all the acquired data to a common FTP server which enabled the annotation of the MSI data based on the optical images in FlexImaging (Bruker Daltonik) by a single center (Fig. 1c).

The bovine serum albumin (BSA) control measurement data was also preprocessed centrally following the description that can be found in the ESM, Protocol S2. Ultimately, the spectra were tested for the presence of nine BSA peptides peaks within a 300-ppm mass error tolerance and a signal-to-noise threshold of 3 to define the lower limit of detection for each peak and dilution.

The MSI data was preprocessed by first recalibrating four datasets in FlexAnalysis due to the presence of mass shifts (ESM, Protocol S3). Once recalibrated, all the nonreduced MSI data was merged in SCiLS Lab (v. 2016b, Bruker Daltonik) for each molecular class separately. During the import, both peptide and metabolite spectra underwent baseline removal with the convolution algorithm (width = 20) and automatic resampling. In SCiLS Lab, all peptide spectra were normalized on the total ion count (TIC) and metabolite spectra on the root mean square value (RMS).

After importing, all the annotated tissue regions were combined and an average tissue spectrum for each molecular class was generated. These overview spectra were then exported for peak picking to mMass (ESM, Table S2) [15]. The detected peaks were re-imported into SCiLS Lab and optimal peak intervals were defined for the peptide (200 ppm) and metabolite datasets (0.15 Da). Finally, the maximum intensity for each peak and tissue core region was exported for all three peak lists into a CSV file for further statistical analysis.

### Data analysis

**Fig. 2:**
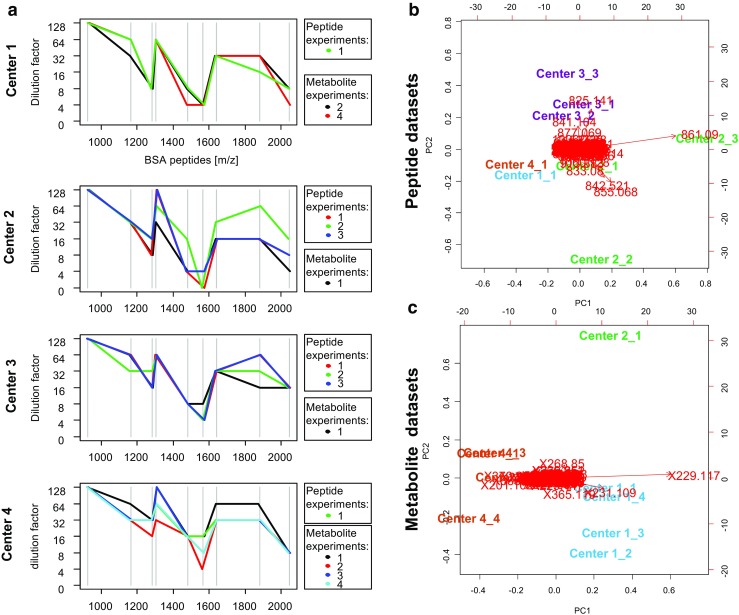
To monitor the instruments’ sensitivity, the lowest limit of detection (=highest dilution factor) was determined for nine bovine serum album (BSA) peptides (vertical gray lines) before any mass spectrometry imaging (MSI) experiment (**a**). These instrument sensitivity profiles were compared to the behavior of the corresponding MSI tissue profiles in the principal component analysis (PCA) space for the detection of potential experimental outliers (**b**, **c**). The PCA plots also show the most influential *m*/*z* signals for each principal component (red arrows) such as *m*/*z* 842.521, which is an autolysis product of trypsin, or *m*/*z* 229.117, which is the matrix 9-aminoacridine

The CSV files were imported into the R statistical environment (v. 3.4.2) [16]. If not mentioned otherwise, standard parameterization was used for all subsequently described methods. The initial principal component analysis was done without scaling to reveal influential mass signals in a biplot (Fig. 2b, c). Afterwards, influential and sample preparation-related peaks were removed whose Pearson correlation coefficients were greater than 0.75 to signals of the trypsin autolysis peptide (*m*/*z* 842.5) or the 9-aminoacridine matrix (*m*/*z* 229.1 = [M+Cl]^−^) for the peptide and metabolite MSI peak lists, respectively (ESM, Table S2).

A structured overview of all subsequently described data analysis methods is shown in ESM, Table S3. Coefficients of variation were calculated based on the estimated variance components yielded by mixed-effect models using the R package ‘lme4’ divided by the mean intensity of each respective peak. In these models, the tissue type was considered a fixed effect and experiment and center random effects. For comparisons within a molecular class, differences in the coefficients of variation between levels (intra- vs. intercenter) were investigated using the sign test. Differences in coefficients of variation between peptides and metabolites were studied using Mann-Whitney *U* test. Univariate statistical testing for finding discriminating masses within centers and between each pair of tissue type was performed using Student’s *t* test followed by Benjamini-Hochberg correction for multiple testing. Center-wide discriminatory power was assessed by meta-analysis via a random-effects model and the standardized mean difference as outcome measure (R package ‘metafor’). For all mentioned tests, *p* values ≤ 0.05 were considered statistically significant.

**Fig. 3:**
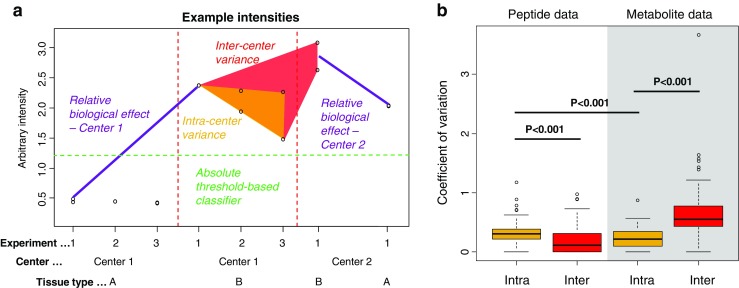
One of the goals of this round robin study is to investigate the effect of technical variance on masking the biological effect between the different organs on the TMA. **a** The difference in detected intensities due to the biological effect (purple lines) and the scattering of the intensities due to intra- (orange polygon) and intercenter (red polygon) technical variance is illustrated. The latter both have been quantified as coefficients of variation for each mass signal and molecular class by a linear mixed-effects model (**b**). These variations might hamper absolute comparisons of intensities, such as the transfer of single-center optimized absolute cutoffs to discriminate tissue types in other centers (green dashed line, **a**)

Univariate classificatory power for each peak to separate two tissue types was evaluated by determining an optimal cutoff value (Fig. 3a) using the CART algorithm in the R package ‘rpart’. To overcome overfitting, the CART model was pruned to have only one branch at the root by setting parameters to: minsplit = 1, maxdepth = 1, minbucket = 1, and cp = 0.001. Supervised multivariate classification was performed using the random forest algorithm (R package ‘randomForest’), which was fed with the 70 most discriminating masses sorted by their *p* values as determined by an upfront analysis of variance.

## Results

For this round robin study, consecutive sections of a formalin-fixed paraffin-embedded tissue microarray (TMA) containing 16 biopsies from eight different mice organs were distributed among the four participants (Fig. 1a). The TMAs were measured in each center on a metabolite and peptide level. The data from all contributors was gathered, annotated, merged, and analyzed centrally (Fig. 1b). Peak picking and subsequent cleanup led to 165 and 189 mass signals in the peptide and metabolite datasets, respectively (ESM, Table S2). Due to a significant core loss of liver and kidney tissues in the peptide experiments, these organs were excluded from further analysis.

### Quality controls and outlier detection

Each MSI experiment was preceded by quality control measurements of a centrally distributed concentration series of BSA peptides. All centers showed similar BSA sensitivity profiles, although with some intracenter variation. We next investigated if these sensitivity profiles can be related to the corresponding MSI peptide and metabolite tissue profiles in the principal components analysis (PCA) space (Fig. 2). The PCA biplot not only shows that peptide measurements 2 and 3 of center 2 are different than the remaining experiments, but also that their dissimilarity is mostly attributed to the variables *m*/*z* 861.1 (matrix cluster: M_4_KNa_3_-H_3_) and *m*/*z* 842.5 (a trypsin autolysis product [17]), and hence not related to the instrument performance but rather to sample preparation. In contrast, the deviation of metabolite experiment 1 of center 2 was not related to any variable in particular since *m/*z 229.1, which is 9-aminoacridine + chloride [18], stands orthogonal to principal component 2, which discriminates this experiment from the rest. The corresponding BSA control measurement preceding metabolite experiment 1 of center 2 (Fig. 2a), however, does not suggest a lower instrument performance. In contrast, metabolite experiment 1 of center 3 is not shown as it was excluded from the analysis due to a wrongly selected instrumental method during acquisition.

The variance-driven PCA analysis also gives an impression of the intra- and intercenter relations and distances of the single experiments and therefore of the variances caused by the intra- and intercenter effects, which can be quantified.

### Quantification of intra- and intercenter variation

One of the goals of this round robin study was to investigate the effect of technical variation on masking the biological effect between the different organs on the TMA. Figure 3a illustrates the difference in detected intensities due to biological effects and the scattering of the intensities due to intra- and intercenter technical variation. Both have been quantified as coefficients of variation for each mass signal and molecular class by a linear mixed-effects model. The results are presented in Fig. 3b which show that for the peptide dataset, the intracenter experimental variation (median = 0.30) of peptides was significantly higher than the intercenter variation of peptides (median = 0.12; *p* < 0.001) and also significantly higher than the intracenter experimental variation of metabolites (median = 0.22; *p* < 0.001). However, the latter was observed to be 2.5 times lower than the intercenter variation of metabolites (median = 0.55; *p* < 0.001). This observation might hamper absolute intercenter comparisons of intensities on a metabolite level.

### Reproducibility of univariate tissue comparisons

**Fig. 4:**
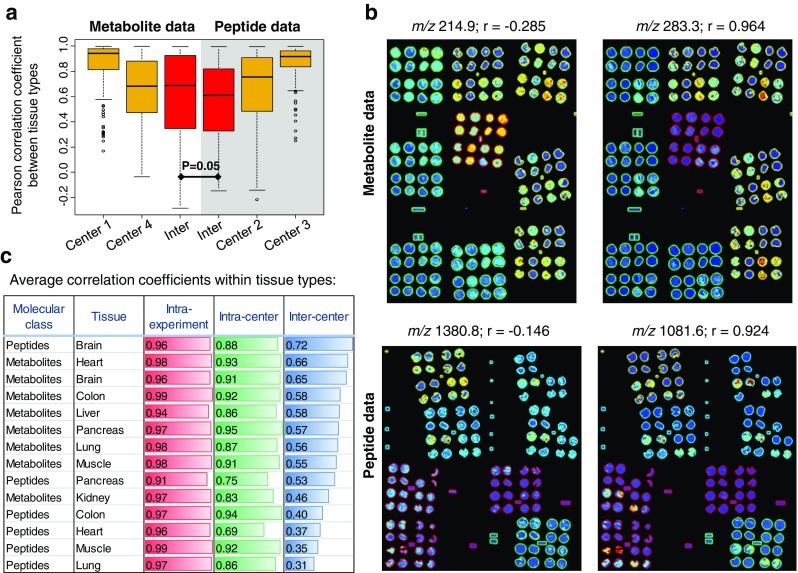
The reproducibility of univariate visualization patterns between tissues and multivariate profiles within a tissue type was investigated using the Pearson correlation coefficient *r* which can quantify the degree of similarity. First, all intracenter and intercenter experiments have been compared pairwise, and the correlation coefficient was calculated for each mass signal, where higher values of *r* indicate a higher reproducibility (**a**). Examples for mass signals with high (right hand side) and low (left hand side) reproducibility are shown (**b**). The reproducibility of multivariate tissue-specific profiles was also investigated within experiments, between experiments and centers (**c**)

**Fig. 5:**
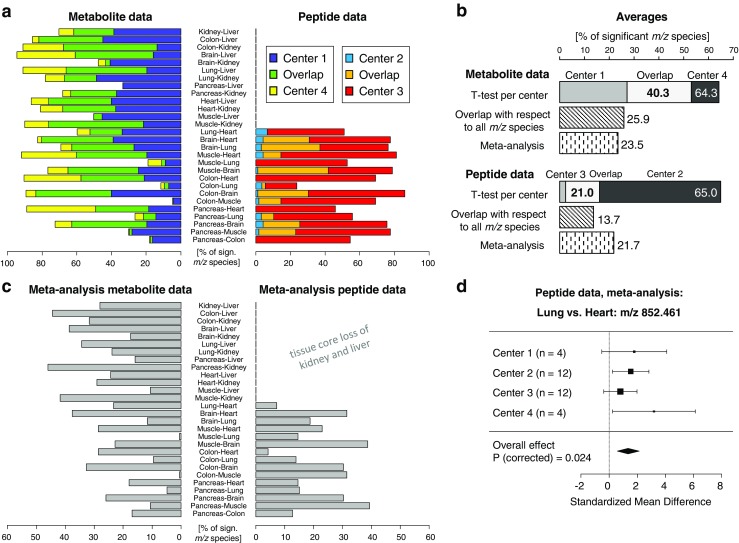
The reproducibility of univariate statistical testing between each pair of tissue type was investigated by comparing the percentage of significant *m*/*z* species found for each center (only centers with a minimum of three experiments were considered) and the overlap between centers (green and orange bars) (**a**). The summary in **b** shows that peptide and metabolite data were overall equally discriminative, but the metabolite data was more reproducible. The meta-analysis results per tissue type comparison are shown in **c**. Especially the peptide data benefitted from the combination of cross-center effects, since it could assemble the samples from four centers (**b**, **d**)

The reproducibility of univariate signals was assessed in two forms: first, by looking at intensity patterns for each mass signal across all tissues within one experiment and compare those visualization patterns within and between centers using the Pearson correlation coefficient *r* (Fig. 4a), and second, by using statistical testing to discriminate pairs of tissue and compare these results within and between centers (Fig. 5). For both approaches, only centers with at least three experiments for each molecular class were considered.

The intensity pattern approach shows that there is a slight advantage of metabolites (median = 0.69) over peptides (median = 0.61) to reproducing intensity patterns between centers (*p* = 0.05), but there is, in both sides, strong center-dependent variation (Fig. 4a). Examples are shown in Fig. 4b.

In the second approach, the reproducibility of statistical testing between each pair of tissue type was investigated by comparing the significant masses found per individual center. Figure 5a shows the percentage of significant variables found for each center and tissue pair comparison separately and the overlap between the two centers. While the discriminatory potential depends on the pair of tissue type (e.g., colon vs. muscle or colon vs. brain), the metabolite data exhibits overall a higher overlap (40. vs. 21.0% overlapping and significant *m*/*z* species) and, therefore, a higher reproducibility of the results across the centers (Fig. 5b).

Meta-analyses, a common statistical approach in intercenter studies, were performed to investigate the increase in statistical power by combining the number of samples and effects from different centers (Fig. 5c). Especially the peptide data benefited from the meta-analysis for detecting biological differences in masses that were otherwise not found in a single-center analysis (13.7 vs. 21.7%; Fig. 5b). An example is shown in Fig. 5d.

### Reproducibility of multivariate tissue profiles

After the univariate analysis of intensity visualizations between tissues, we also investigated the multivariate reproducibility of molecular patterns of each individual tissue type. This was done by calculating the Pearson correlation coefficient for each tissue type separately between the spectra from within one experiment, between experiments, and between centers. The results are shown in Fig. 4c. It can be seen that there are differences with respect to the tissue type but also with respect to the molecular class. For instance, peptides and metabolites agree on that muscle tissue shows lower reproducibility than the brain whereas heart tissue ranks average for reproducibility in peptides but high in metabolites. Please note that the correlation coefficient is insensitive to additive or multiplicative effects between spectra, and evaluates the relative relationship between data points as compared to the coefficients of variation in Fig. 3b, which capture more absolute effects.

### Univariate vs. multivariate supervised classification

**Fig. 6:**
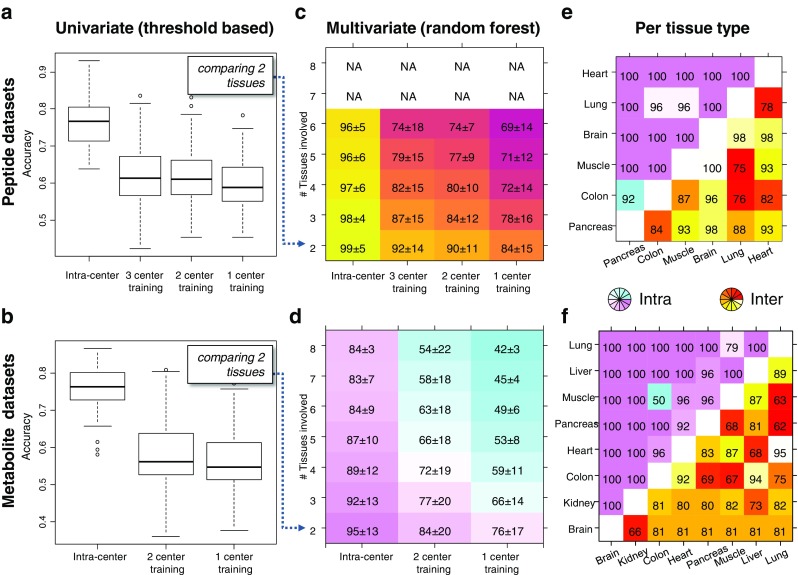
The performance of uni- and multivariate classifiers between centers was investigated by moving centers continuously from the training to the test set. Univariate classifiers were built for each pair of tissue and *m*/*z* species by determining an optimal intensity threshold in the training set (Fig. 3a) and were evaluated on the test set. The observed accuracies are reported in **a** and **b**, where the intracenter accuracies served as reference. The approach was extended to all tissue types and the usage of multivariate patterns employing the random forest algorithm. **c**, **d** The mean accuracy [%] and standard deviation as a function of number of tissue types involved and the number of centers in the training set. **e**, **f** The accuracies [%] for each pair of tissues for intra- and intercenter classifications

Next, it was examined if the molecular discriminatory information for distinguishing two tissue types can be directly transferred between centers; a schematic is shown in Fig. 3a. This was done by optimizing a threshold for each *m*/*z species* in the training set using a CART model followed by its application to a test set. It was then determined how the cross-center performance of the classifier changes with the amount of training data by continuously moving centers from the training to the validation set. The intracenter accuracies were therefore calculated as reference and their means were for both peptides and metabolites 76% (Fig. 6a, b). When applying these threshold-based classifiers to the data from other centers, significant drops in accuracies were observed: − 15 and − 18 percentage points (ppts) for peptides and metabolites, respectively, when looking at two center training.

Next, it was explored if classifiers based on a multivariate signature would be more robust to classify data across different centers. Therefore, a random forest classifier was used, as it automatically performs a feature weighing, and intracenter accuracies were calculated as reference. The mean accuracy for the peptide data ranged from 92% (three center training, two tissue types) to 84% (one center training, two tissue types) and from 74 to 69% (for all six tissue types; Fig. 6c). These results show a beneficial effect of having more training data in order to cope with center-related noise in the data and an increase in difficulty when dealing with a rising number of classes. The mean metabolite accuracies ranged from 84% (two center training, two tissue types) to 76% (one center training, two tissue types) (Fig. 6d) and were hence 6–8 ppts lower than the peptide data for classifying two tissue types and up to − 20 ppts less accurate when classifying six tissue types. The performance also depends on the detectable degree of chemical difference between each pair of tissues, which are shown for intra- and intercenter comparisons and for peptides and metabolites separately in Fig. 6e, f. It can be recognized that certain tissues can be more accurately separated by certain molecular classes, such as the pancreas/lung by peptides and heart/lung by metabolites.

### Comparison of normalization methods

**Fig. 7:**
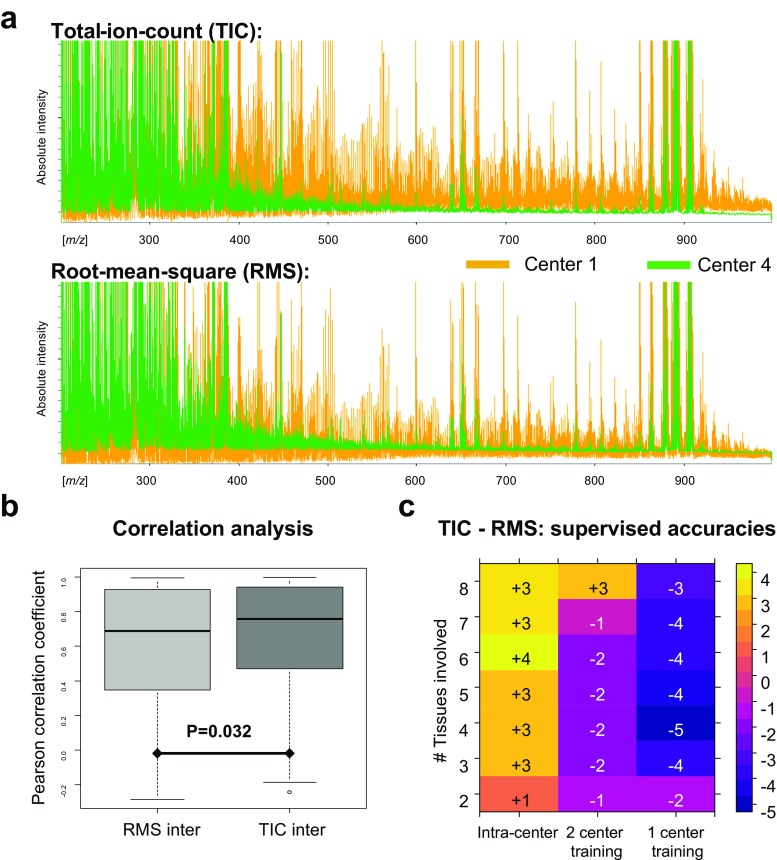
Total ion count (TIC) and root mean square (RMS) are commonly used normalization methods in mass spectrometry imaging for metabolite data. **a** The effect of the two normalization methods on the spectral baselines of each center where the baselines from the two centers seem to move toward each other when using RMS normalization. This is also reflected in the performance of multivariate classifiers where RMS outperformed TIC normalization for intercenter comparisons and vice versa for intracenter comparisons (**c**). For relative intercenter comparisons as performed by the correlation analysis, TIC outperforms RMS (**b**)

Normalization of the spectral data is a crucial step for comparisons between MSI datasets. While the TIC is the gold standard for peptide, protein, and lipid MSI datasets measured with time-of-flight-based mass analyzers (as used here), for metabolite MSI datasets, there is no gold standard yet. In this work, RMS was used but the TIC has also been used by others [19]. It was therefore investigated which of the normalization strategies enable a better comparability between the different metabolite datasets. The consequences on spectral level are depicted in Fig. 7a where the baselines of both centers clearly move toward each other with the RMS normalization. The effect of the spectral displacement was evaluated on a univariate and multivariate level. With respect to the first, the overall observation was that the TIC normalization leads to an improvement of relative intercenter comparisons of intensity patterns (Fig. 7b). The multivariate classification, as absolute intensity-based approach, showed that the RMS normalization showed a better multivariate performance across centers, whereas TIC was favorable for intracenter comparisons (Fig. 7c).

## Discussion

Multicenter or round robin studies are important for developing optimal standards and protocols that ensure sufficient high sensitivity, specificity, and reproducibility of experiments between centers. Ultimately, a high degree of comparability is a necessity for multicenter clinical studies. This has already been recognized by several multicenter initiatives in the field of mass spectrometry, such as the Clinical Proteomic Technology Assessment for Cancer (CPTAC) network [20], the Spanish network of proteomics laboratories (ProteoRed-ISCIII) [11, 21], or several MALDI-Biotyper ring trials (ESM, Table S4).

In line with these efforts in mass spectrometry, we present here the results of the first round robin study in MSI on formalin-fixed paraffin-embedded tissues. A minimum of four samples distributed over two molecular classes have been analyzed by four centers, which is comparable to other non-LC/MS ring trials in terms of number of centers and number of replicates (ESM, Table S4), with the aim to assess relative and absolute reproducibility between centers for peptides and metabolites on a uni- and multivariate level. An overview of all data analysis methods used in this study is given in ESM, Table S3. The term *relative* describes comparisons of biological effects that are detected on each center’s own intensity scale (Fig. 3a). In mass spectrometry imaging, all reported results so far from multicenter studies were based on the reproducibility of relative effects [6, 7], except the study by Abbassi-Ghadi et al. who looked at the variation of lipid signal intensities in desorption electrospray ionization MSI experiments between two laboratories [12].

Here, when investigating relative univariate effects, it turned out that the metabolite data exhibited an overall higher overlap of the results across the centers, compared to the peptide data (Figs. 4c and 5). An explanation could be that the intracenter variation of center 2 for the peptide data is already high compared to center 3, as can be deduced from the PCA plot (Fig. 2b), which is confirmed by the analysis of variance which shows that the intracenter variation of peptide data is significantly higher than in the metabolite data (Fig. 3b). But statistical significance of a biological phenomenon not only depends on the interplay of detectable biological effects and technical variance but also on the number of samples involved. The latter might benefit from the higher number of samples offered by merging intercenter data through a meta-analysis. Especially the peptide dataset benefited from the meta-analysis, as it could assemble the samples from four centers compared to the metabolite data with only three centers, which led to a 1.5 times increase in the detection of biological differences (Fig. 5b). This suggests that meta-analysis may be a powerful solution to increase sensitivity for the discovery of relative, but still generally valid biomarkers.

While a meta-analysis combines relative effects between centers, *absolute effects* are effects that can be directly transferred between centers such as intensity cutoffs for classification (Fig. 3a). As absolute effects share the same intensity scale, it is important to quantify the additional variation caused by intercenter comparisons. In this study, we observed the metabolite data to suffer from a significantly higher intercenter experimental variation compared to its intracenter variation, whereas this observation was vice versa in the peptide data (Fig. 3b). However, the combined intra- and intercenter technical variances had similar unfavorable consequences on the performance of univariate classifiers between centers for both molecular classes (Fig. 6a, b).

In contrast, the multivariate approach outperformed the univariate approach on average by more than 25 ppts (Fig. 6c, d). It can also be seen that the more centers were involved in the training of the classifier, the better the prediction. This shows that a multivariate classifier can learn to extract the relevant information from intercenter noise. It was found that the optimum molecular class for differentiating tissue types was tissue type dependent (Fig. 6e, f) and that multivariate classifiers based on peptides were in general observed to be more accurate for intercenter comparisons (Fig. 6). This is unforeseen, since the sample preparation for the detection of peptides contains two additional and relatively intensive steps (antigen retrieval and on-tissue digestion), both of which were expected to increase the technical variance between centers. This observation requires further investigation.

On the other hand, the lower performance of the metabolite data in the multivariate classification can be ascribed to the higher intercenter variation which might be also related to the nonoptimal equalization of the baselines in time-of-flight instruments (Fig. 7a). Laser intensity is a crucial parameter to influence the baseline, which was left undefined and therefore to be optimized freely according to the local experimenter’s subjective opinion on the quality of the spectra. To objectivize, a laser power meter might be recommendable to match laser intensities between centers [22]. At this stage, software normalization is the only way to compensate these differences.

So far, our observations indicate that RMS normalization is more beneficial for absolute intercenter comparisons and TIC normalization for relative inter- or intracenter comparisons. Alternative normalization methods are hence needed, as they have already been proposed for protein MSI datasets [23].

Also, further investigations have to be performed on multicenter studies with more and other tissues since the biological differences studied here are not representative for most of the biomedical research questions such as tumor biomarkers. The aim of this study was to make the first step toward multicenter studies involving FFPE tissues. We strongly recommend future studies to further develop methods to monitor instrument performance, as done here, but also to monitor the sample preparation, since some of the intracenter variance-inducing effects could be ascribed to sample preparation, such as matrix- and digestion-related effects as deduced from the PCA biplot (Fig. 2b). For on-tissue digestion, such quality controls have already been proposed [24] but are still missing for the matrix application.

Altogether, in the light of the results of this study combined with new quality controls for sample preparation and novel normalization methods, we foresee a high potential for running successfully multicenter mass spectrometry imaging studies on FFPE samples.

## Electronic supplementary material

ESM 1 (PDF 276 kb)
