# Supplementary material for: Round robin study of formalin-fixed paraffin-embedded tissues in mass spectrometry imaging
Source: Anal Bioanal Chem. 2018 Jul 3;410(23):5969–80. doi: 10.1007/s00216-018-1216-2 (PMC6096706; doi:10.1007/s00216-018-1216-2)
Supplement: Supplementary file 1 — (PDF 276 kb) [file 216_2018_1216_MOESM1_ESM.pdf]

Analytical and Bioanalytical Chemistry

Electronic Supplementary Material

Round Robin Study of Formalin-Fixed Paraffin-Embedded Tissues in Mass Spectrometry Imaging

Achim Buck, Bram Heijs, Birte Beine, Jan Schepers, Alberto Cassese, Ron M.A. Heeren, Liam A. McDonnell, Corinna Henkel, Axel Walch, Benjamin Balluff

Protocol S1: BSA concentration series preparation

1 nmol bovine serum albumin standard (Pierce™ BSA Protein Digest, # 88341, Thermo Fisher) was mixed with 500 µL of a 0.2% trifluoroacetic acid (TFA) solution to a starting concentration of 2 µMolar. Then it was six times continuously diluted 1:1 with 0.2 % TFA. This concentration series was shipped to all partners on dry ice. Each center mixed each dilution 1:1 by with the alpha-cyano-4-hydroxycinnamic acid (CHCA) matrix solution (7 mg/mL CHCA in 50 % ACN/0.2 % TFA). Finally, 2 µL are spotted for each concentration on a previously cleaned AnchorChip target:

		**Number of dilution steps and resulting concentration [Molar]**					
**Dilution factor with 0.2 % TFA**	**Dilution factor with CHCA**	**1**	**2**	**3**	**4**	**5**	**6**
**2**	**2**	5E-07	2.5E-07	1.25E-07	6.25E-08	3.13E-08	1.56E-08
**Starting conc. [Molar]**	**Spotted volume [µL]**	**Absolute amount in spotted volume [mol]**					
0.000002	2	1E-12	5E-13	2.5E-13	1.25E-13	6.25E-14	3.13E-14

Protocol S2: BSA data processing

In order to enable a better comparability, all submitted bovine serum albumin (BSA) spectra were re-processed with the same FlexAnalysis (Bruker Daltonik, Bremen, Germany) method, consisting of the following commands and parameters: UndoAllProcessing; ApplyRawCalibration; faSmooth(Gauss: width=0.02 m/z; cycles=2); faSubtractBaseline (TopHat); faFindPeaks (SNAP: minimum S/N=3; MaximumNumberOfPeaks=300; Averagine).

Then the data underwent a rough internal re-calibration in FlexAnalysis with 1500 ppm mass error tolerance on the following *m/z* signals: *m/z* 927.4934 (BSA peptide), *m/z* 1554.655 (omni-present mass signal), and *m/z* 1749.676 (omni-present mass signal).

Finally, the BSA peak detection was performed by looking for 9 BSA peptides (*m/z* 927.4934, 1163.631, 1283.711, 1305.716, 1479.795, 1567.743, 1639.938, 1880.921, and 2045.028) within a 300 ppm mass error tolerance. The lower limit of detection of each peptide can be then plotted in dependence of the dilution factor, as shown in Figure 2a.

Protocol S3: MSI data pre-processing

For a better comparability of the spectral signals, all metabolite datasets of center 4 underwent recalibration. This was done in a two-step approach by first recalibrating roughly on two matrix peaks (*m/z* 229.15 and 385.23 with tolerances 7,000 and 5,000 ppm, respectively) and in a second round more precisely (tolerance: 1000 ppm) on three peaks (by adding the lipid peak *m/z* 888.7). All of this was done in FlexAnalysis (Bruker) in a batch run with the following processing parameters: UndoAllProcessing; ApplyRawCalibration; faFindPeaks (Centroid: RelativeIntensityThreshold=10%; MaximumNumberOfPeaks=30; PeakWidth=*m/z* 0.2; Height=80%); CalibrateInternal.

The peptide dataset of center 1 was recalibrated by copying the calibration constants from one a manually calibrated spectrum to the remaining dataset using the batch function “ReplaceCalibration” of FlexAnalysis (Bruker). The manual calibration was based on 4 peptide signals *m/z* 842.512 (trypsin), *m/z* 944.578 (histone H4), and two other peptide signals at *m/z* 1551.846 and 2141.188.

Table S1: Chemicals

	**Center 1**	**Center 2**	**Center 3**	**Center 4**
Xylene	Xylene, 99%, Carl Roth #9713	Roth, Xylol (Isomere), ≥98,5 %, Ph.Helv., #CN80.2	Xylene Histograde, J.T. Baker #3410	Honeywell Xylenes, 99%, #16446
9-aminoacridine hydrochloride monohydrate	9-Aminoacridine hydrochloride monohydrate, 98%, Sigma-Aldrich #A38401	9-Aminoacridine hydrochloride monohydrate, 98%, Aldrich #A38401	9-Aminoacridine hydrochloride monohydrate, 98%, Aldrich #A38401	9-Aminoacridine hydrochloride monohydrate, 98%, Aldrich #A38401
Citric acid	Citric acid, 99.0%, Sigma-Aldrich #C0759	Citric acid monohydrate, ACS reagent, ≥99.0%, Sigma-Aldrich #C1909	Citric acid monohydrate, ACS reagent, ≥99.0%, Sigma-Aldrich #C1909	Citric acid monohydrate, ACS reagent, ≥99.0%, Sigma-Aldrich #C1909
Trypsin	Trypsin Gold, mass spectrometry grade, Promega #V5280	Trypsin Gold, mass spectrometry grade, Promega #V5280	Trypsin Gold, mass spectrometry grade, Promega #V5280	Trypsin Gold, mass spectrometry grade, Promega #V5280
alpha-Cyano-4-hydroxycinnamic acid	α-Cyano-4-hydroxycinnamic acid, ≥99%, Sigma-Aldrich #70990	α-Cyano-4-hydroxycinnamic acid, Bruker #201344	α-Cyano-4-hydroxycinnamic acid, ≥98%, Aldrich #476870	α-Cyano-4-hydroxycinnamic acid, ≥98%, Sigma-Aldrich #C2020
Trifluoroacetic acid	Trifluoroacetic acid, HPLC grade, Applied Biosystems #400445	AlfaAesar, Trifluoroacetic acid, HPLC grade, 99,5+%, #44630	Trifluoroacetic acid, LC-MS Ultra, Fluka #14264-2ML	Trifluoroacetic acid, HPLC grade, ≥99.0%, Sigma-Aldrich #302031-M

Table S2: mMass peak picking parameters

			**Peptide average spectrum**	**Metabolite average spectrum**
**SCiLS parameters**				
	Normalization		**TIC**	**RMS and TIC**
	Mass window		200 ppm	0.15 Da
**mMass parameters**				
	Baseline correction	Precision	35	35
		Relative offset	0	0
	Smoothing	Method	-	Savitzky-Golay
		Window	-	0.2
		Cycles	-	2
	Peak picking	S/N	3	5
		Picking height	77	100
	De-isotoping	Max. charge	1	1
		Isotope mass tolerance	0.2	0.2
		Isotope intensity tolerance	50	50
	Peak picking results	Number of peaks	169	201 and 192
**R mass filtering**	Reference masses	Trypsin autolysis peak	*m/z* 842.5	-
		[9-aminoacridine matrix + Cl]-	-	*m/z* 229.1
	Pearson correlation coefficients	Minimum correlation	0.75	0.75
	Clean-up results	Number of peaks	165	194 and 189

Table S3: Overview of data analysis types

<table>
<tr><td colspan="2"></td><td colspan="2">Analysis type</td></tr>
<tr><td></td><td></td><td>Relative (each center with own intensity scale)</td><td>Absolute (centers share intensity scales)</td></tr>
<tr><td rowspan="4">Number of m/z species involved</td><td rowspan="2">1 (univariate)</td><td>Pearson correlation analysis of visualization patterns between tissues (Fig. 4a,b)</td><td>Coefficient of Variation analysis (Fig. 3b)</td></tr>
<tr><td>Comparison of peak lists after statistical testing between tissues (Fig. 5a,b)</td><td>Threshold based classifier: CART (Fig. 5a,b)</td></tr>
<tr><td rowspan="2">>1 (multivariate)</td><td rowspan="2">Pearson correlation analysis of molecular patterns within tissues across experiments and centers (Fig. 4c)</td><td>Principal Component Analysis (Fig. 2b,c)</td></tr>
<tr><td>Classification: Random Forest (Fig. 5c,d,e,f)</td></tr>
</table>

Table S4: References on non-LC/MS ring trials

Publication title	Reference	Technology	Number of centers	Number of replicates
Multicenter Matrix-Assisted Laser Desorption Ionization–Time of Flight Mass Spectrometry Study for Identification of Clinically Relevant *Nocardia spp*	Blosser SJ et al., J Clin Microbiol. 2016 May; 54(5): 1251–1258	MALDI Biotyper	3	On average 28.3 repeated extractions
A multi-center ring trial for the identification of anaerobic bacteria using MALDI-TOF MS	Veloo ACM et al., Anaerobe. 2017 Dec;48:94-97	MALDI Biotyper	7	Not reported
Multicenter Evaluation of the Bruker MALDI Biotyper CA System for the Identification of Clinically Important Bacteria and Yeasts	Wilson DA et al., Am J Clin Pathol. 2017 Jun 1;147(6):623-631	MALDI Biotyper	6	Maximum 10
Multicenter Evaluation of the Bruker MALDI Biotyper CA System for the Identification of Clinical Aerobic Gram-Negative Bacterial Isolates	Faron ML et al., PLoS One. 2015 Nov 3;10(11):e0141350	MALDI Biotyper	5	Repeated only if score was too low
Repeatability and reproducibility of desorption electrospray ionization-mass spectrometry (DESI-MS) for the imaging analysis of human cancer tissue: a gateway for clinical applications	Abbassi-Ghadi N et al., Analytical Methods. 2015;7(1):71-80	DESI	2	4
Analysis of Human Proteome Organization Plasma Proteome Project (HUPO PPP) reference specimens using surface enhanced laser desorption/ionization-time of flight (SELDI-TOF) mass spectrometry: Multi-institution correlation of spectra and identification of biomarkers	Rai AJ et al., Proteomics 2005, 5, 3467–3474	SELDI	5	Minimum 2
